# Effects of a participatory work stress prevention approach for employees in primary education: results of a quasi-experimental study

**DOI:** 10.5271/sjweh.4141

**Published:** 2024-04-01

**Authors:** Maartje C Bakhuys Roozeboom, Noortje M Wiezer, Roosmarijn MC Schelvis, Irene MW Niks, Cécile RL Boot

**Affiliations:** 1 Netherlands Organisation for Applied Scientific Research TNO, Unit Healthy Living & Work, Leiden, The Netherlands.; 2 Body@Work, Research Center on Work, Health and Technology, TNO/VUmc, Amsterdam, The Netherlands.; 3 Amsterdam public health research institute, Societal participation and Health, Amsterdam, the Netherlands.; 4 Amsterdam UMC location Vrije Universiteit Amsterdam, Public and Occupational Health, Amsterdam, The Netherlands.; 5 Amsterdam UMC, location University of Amsterdam, Public and Occupational Health, Amsterdam, The Netherlands.

**Keywords:** effect evaluation, organizational-level occupational health intervention

## Abstract

**Objective:**

Work stress is a serious problem for employees in primary education. This study evaluates the effects of a work stress prevention approach on emotional exhaustion and work stress determinants (job crafting behavior, quantitative and emotional demands, leadership, support, autonomy, team culture and feelings of competence), and the impact of implementation success (management commitment, employee involvement, communication during implementation) on these outcomes.

**Methods:**

A quasi-experimental study was conducted with an intervention group (4 schools, N=102 employees) and a control group (26 schools, N=656 employees) using questionnaires at baseline (T0), one-year (T1) and two-year (T2) follow-up. Multilevel mixed model analyses were performed to test effects of condition and implementation success on changes in emotional exhaustion and work stress determinants between T0 and T2 in the intervention and control group.

**Results:**

No effect were found for emotional exhaustion. Improvement of quality of leadership between T0 and T2 was significantly larger in the intervention compared to the control group. Additionally, implementation success was associated with a decrease in unnecessary demands and an increase in quality of leadership, team culture and job crafting behavior.

**Conclusions:**

This study shows no direct effect of the approach on emotional exhaustion, but it does show beneficial effects on quality of leadership. Additionally, results suggest that, when successfully implemented, the approach also has beneficial effects on other work stress determinants (ie, job crafting behavior, unnecessary demands and team culture). Results indicate that – if implemented successfully – the organizational-level intervention has the potential to improve the psychosocial work context.

Work stress is an urgent issue among workplaces around the globe that can lead to work-related emotional exhaustion. Especially in education, the number of employees reporting work-related emotional exhaustion is high (1), and this can have severe consequences on teachers’ health, students and schools (2). Effective interventions are badly needed. Over the past decades research has provided evidence for the importance of interventions to help teachers cope with stressors (3, 4). However, a problem with these kind of interventions is that they do not focus on the underlying source of the problem (5). Organizational-level occupational health interventions however do focus on reducing the causes of work stress (6). During these interventions work stress determinants are identified and tailored actions are implemented to mitigate or remove these determinants. These interventions are characterized by employee participation during all steps of the approach, which is believed to empower employees to improve their working conditions (7, 8) and secures that planned actions fit in with the organizational culture (9, 10). Although these interventions are considered the gold standard (11–13) – and there is evidence for their effectiveness (14) – in practice, they often do not bring about the intended outcomes (15).

A possible explanation for this is the selection of inappropriate actions (ie, actions that do not consist of the effective ingredients to decrease work stress determinants) (16). Ensuring the appropriateness of actions, requires a theory of change (17). In contrast to the abundance of theories linking determinants to health outcomes (eg, work stress), theories linking planned actions to changes in determinants are scarce and seldomly used in organizational-level interventions (6, 18). Therefore, building a logic model of change could be of added value to the work stress prevention approach because it maps the program logic: What needs to change to reduce work stress? What determinants should the measures target? What actions are appropriate to affect the determinants? Answering these questions provides guidance for selecting appropriate actions (19, 20) that can be implemented successfully (21).

Another explanation for organizational-level occupational health interventions not bringing about the intended results is the unsuccessful implementation of the actions (22). Previous research on the application of a similar approach in primary education showed that the implementation of the action plans phase is particularly important, whereas especially during this phase it is difficult to keep employees informed and involved and managers committed (23). Providing feedback on factors that can hinder or facilitate the implementation such as management commitment, employee involvement, and communication (22) could provide the opportunity for implementors to act on hinderances the moment they occur and may reduce the risk of implementation failure (24, 25).

The focus of the current study is an organizational-level occupational health intervention (ie, work stress prevention approach) for primary education. To ensure the selection of appropriate measures and decrease the risk of implementation failure, in the current study this approach is expanded with (i) building a logic model of change to facilitate action planning and (ii) real-time feedback of the implementation process to implementers to prevent implementation failure.

Organizational-level interventions are challenging to evaluate and traditional randomized controlled trial designs often do not match with the dynamics of the organizational context that is hard to control (26). To provide more information on intervention effects in relation to implementation success, several researchers have proposed to use data from the evaluation of the implementation process in the effect evaluation (23, 26–28). They suggest to use data on implementation factors eg, management commitment, employee involvement, and communication as a proxy for the level of implementation, and investigate whether this impacted changes between baseline and follow-up on the outcome measures.

This paper aims to evaluate the effects of this work stress prevention approach that was implemented in primary education workplaces in The Netherlands. The following research questions (RQ) were formulated: To what extent did the work stress prevention approach in intervention schools reduce emotional exhaustion over a two-year follow up period, compared to control schools (RQ1)? To what extent did the work stress prevention approach in intervention schools change work stress determinants over a two-year follow up period, compared to control schools (RQ2)?

In addition, RQ were formulated to test whether the implementation process impacted effects of the work stress prevention approach on work stress and work stress determinants: To what extent is there an association between the level of implementation and effects of the work stress prevention approach on emotional exhaustion between baseline and two-year follow up (RQ3)? To what extent is there an association between the level of implementation and effects of the work stress prevention approach on work stress determinants between baseline and two-year follow up (RQ4)?

## Methods

### Study design and study population

In The Netherlands, primary schools generally fall under the governance of larger foundations that provide staff services such as HR practices, personnel recruitment and professional education. Schools each have their own location and can be seen as separate, independent units. This study was initiated by two school foundations and a large research institute in The Netherlands. A total of 30 primary schools (each with 10–35 employees) fell under the scope of these two school foundations. In total, four schools (one small and one large school from each school foundation) could participate in the intervention group. Schools were recruited to participate as intervention school via an email sent out by the school foundations to all school principals. Schools that applied were in fact a large and a small school from each school foundation, and after their application the recruitment procedure was closed. Reasons for participation were, amongst others, signals of work stress reported by employees. All other 26 schools were appointed as control schools. During the intervention, the heads of the intervention schools were asked not to discuss the progress of the intervention with the heads of the control schools. Teaching and non-teaching employees of all schools were invited to participate in the study. Informed consent was obtained from all individual participants included in the study. The Medical Ethics Committee of the VU University Medical Centre (Amsterdam, The Netherlands) approved the study protocol.

### Work stress prevention approach

**Figure 1:**
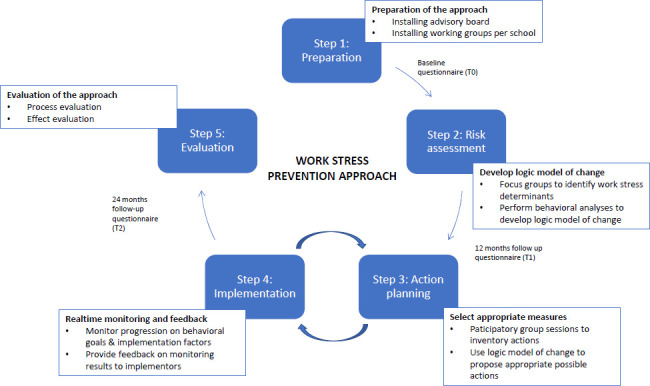
Work stress prevention approach lasting three years in total.

The full program of the approach has been described previously (29). Figure 1 provides an overview of the steps. During step 1, at each school a working group was formed (consisted of the school principal and 2– 3 employees) that was responsible for action planning (step 3) and implementation (step 4). During step 2, work stress determinants were identified by a risk assessment. During step 3, a logic model of change was built by the researchers based on Intervention Mapping (19), by: (i) setting a program objective, (ii) identifying performance objectives (behavioral actions needed to accomplish the program objective), (iii) identifying determinants for the performance objectives and (iv) selecting behavioral change methods to target the determinants. Based on this logic model of change, possible actions were inventoried by participatory group sessions with all personnel and translated by working groups into school specific action plans. Table 1 provides an overview of the results of the risk assessment translated into actions (logic model of change). During step 4, action plans were implemented by the working groups and monthly pulse surveys were carried out among all employees of the intervention schools, measuring the implementation process, progression on determinants and outcomes. Results at school level were fed back to working groups to optimize implementation and/or (further) tailor the action plan if needed. Step 5 consisted of the evaluation, which is the focus of the present study.

**Table 1:** Results of risk assessment translated into measures.

Program goal	Work stress determinants		Behavioral change methods	Measures
	Performance objectives	Determinants based on risk assessment		
Reduce work stress	Manage workload (job crafting behavior; prioritize and adjust tasks, communicate needs, signal overload, set goals)	Job demands (quantitative demands, emotional demands, unnecessary work tasks)	Job re-design	Reduce overlap in administrative tasks; Redivide tasks based on competencies
		Organizational resources (leadership, autonomy, safe team culture, social support)	Social support, modelling, teambuilding	New format performance reviews with principal; Teambuilding activities (organizing sport activities; giving compliment to colleagues) Peer consultation
		Personal factors (feelings of competence)	Self-monitoring, active learning	Individual coaching Training to communicate with parents Monitoring behavioral goals

Employees of the intervention schools took part in the work stress prevention approach lasting three years, whereas employees of the control schools only participated in the baseline and follow-up measurements. Although these steps were similar for all intervention schools, the schools differed regarding the planned actions. In this effect evaluation we intend to study the effects of the approach as a whole.

### Sample size

Sample size was calculated according to the number of cases needed for the effect evaluation of the approach on emotional exhaustion, including two groups with respectively 4 (intervention) and 26 (control) clusters. Due to practical and budgetary constraints, 4 schools could be included in the intervention group. The estimated average cluster size was 15 participants (intervention schools: N=60, control schools N=390). Assuming a significance level (α) of 0.05, two-sided tests and power (1-β) of 0.80 and an intraclass correlation coefficient for schools of 0.01, an effect on emotional exhaustion of Cohen’s d=0.43 could be detected. A review on burnout prevention programs found effect sizes on emotional exhaustion between d=0.29 and d=1.2 (30). This suggests that the anticipated sample size is sufficient to detect an effect on emotional exhaustion.

### Measures

Emotional exhaustion was measured with 5 items of the Utrecht Burnout Scale (UBOS) (31) based on the Maslach Burnout Inventory-General Survey (MBI-GS) (32). The selected subset of items primarily measures the emotional exhaustion component of burnout complaints (eg, I feel emotionally exhausted by my work). Response scales range from 1 = never to 7 = every day (α=0.87).

Job crafting behavior was measured by 6 items selected from the Job Crafting Scale (JCS) (33; eg, I make sure that I make optimal use of my capacities). Response scales range from 1 = totally disagree to 5 = totally agree (α=0.77).

Quantitative demands were measured by 3 items based on the Dutch version of the Job Content Questionnaire (JCQ) (34, 35; eg, Do you have a lot of work to do?) Response scales range from 1 = never to 4 = always (α=0.78).

Emotional demands were measured by 3 items based on the Copenhagen Psychosocial Questionnaire (36; Does your work put you in emotionally disturbing situations?) eg, Response scales range from 1 = never to 4 = always (α=0.74).

Unnecessary work tasks were measured by 4 items based on The Danish Psychosocial Work Environment Questionnaire (DPQ) (37; eg, Do you spend time on work tasks that you have difficulty seeing the purpose of). Response scales range from 1 = to a very large extent to 5 = to a very small extent (α=0.81).

Autonomy was measured by 3 items based on the Dutch version of the JCQ (34, 35; eg, Can you decide for yourself how you do your work?). Response scales range from 1 = yes regularly to 3 = no (α=0.69).

Co-worker support is measured by 3 items of the Dutch ‘Weerbaarheidsmonitor’ (38). The items are originally based on the Dutch ‘Moreelsvragenlijst van Defensie’ (39). Items are slightly adjusted to reflect the work context (eg, I can rely on my colleagues in difficult times). Response scales range from 1 = totally disagree to 5 = totally agree (α=0.92).

Leadership is measured by two scales. Quality of leadership is measured by 4 items based on the DPQ (37; eg, Does your immediate supervisor give high priority to the wellbeing of employees in the workplace?), Response scales range from 1 = to a very large extent to 5 = to a very small extent (α=0.87). Participatory leadership is measured by 4 items of the Dutch ‘Weerbaarheidsmonitor’ (38; eg, The one who supervises me lets me have a say in things that have to do with my work). Response scales range from 1 = totally disagree to 5 = totally agree (α=0.83).

Safe team culture is measured by 3 items from the Dutch ‘Weerbaarheidsmonitor’ (38). The items are based on the Psychological Safety Scale (40; eg, Employees in our team can be vulnerable). Response scales range from 1 = totally disagree to 5 = totally agree (α=0.85).

Feelings of competence is measured by 2 items based on the Basic Needs Satisfaction at Work Scale (41–43; eg, I do not feel very competent when I am at work). Response scales range from 1 = not at all true to 7 = very true (α=0.81).

Implementation process (level of implementation) is measured with 7 items based on the IPM-Q (44) on information (I am aware of the objectives of the approach), communication (I was informed about the progress of the approach), team commitment (I have the feeling that the team is positive about the approach), management commitment (I have the feeling that the principal is positive about the approach), employee involvement (I was involved in the approach), participation in decision making (I could think along with the actions or changes that were implemented as part of the approach), implemented actions (I noticed actions or changes being implemented as part of the approach), that were constructed into a scale. Response scales range from 1 = totally disagree to 5 = totally agree (α=0.90).

Data on potential confounders or effect modifiers were collected at baseline, including age (in years), gender (male, female, other), contract size (number of working hours per week according to contract), function (teacher vs other), job tenure (in years), type of contract [permanent vs temporary (eg, fixed contract, on-call or substitute worker)].

### Statistical analyses

To study effects of the work stress prevention approach multilevel mixed model analyses were performed to adjust for clustering of schools using SPSS version 25 (IBM Corp, Armonk, NY, USA). For all analyses, a value of P<0.05 was indicated as statistically significant. Covariates to include in the analyses were selected based on the “change-in-estimate” approach. In this approach covariate selection decisions are made based upon whether inclusion of a covariate changes the estimate of the causal effect for the exposure with ≥10%. Additionally, based on forward selection covariates were added to the model starting with the covariates that changed the estimate of the causal effect for the exposure the most. Based on this approach the covariates age, contract size and function were included in the analyses.

To investigate RQ1 and RQ2, multivariate mixed model analyses were carried out for emotional exhaustion and work stress determinants with time (T0, T1, T2) and time×condition (intervention versus control) as independent variables. To investigate RQ3 and RQ4, multivariate mixed model analyses were carried out for emotional exhaustion and work stress determinants with time (T0, T1, T2) and time×implementation process as independent variables. In these analyses, the control group received the minimum score on the implementation process scale (score=1). The mixed model analysis method is robust against missing data in the dependent variable because, for maximum likelihood estimations, all observed data in the outcome are used to obtain the parameter estimates for the model.

## Results

### Participant flow

**Figure 2:**
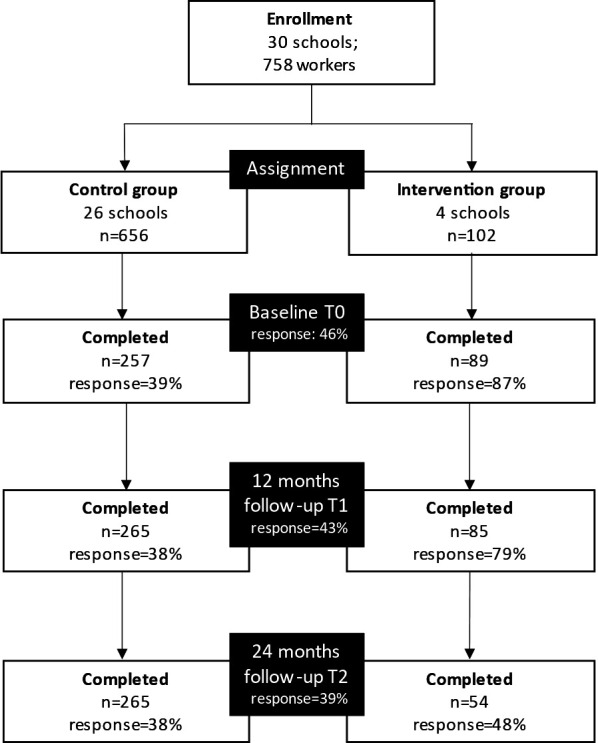
Participant flow of the study.

Since the approach was expected to have an effect at school level, data from new respondents at T1 and T2 were included in the analyses. Figure 2 outlines the participants flow.

### Descriptive statistics

Table 2 shows the baseline characteristics of the study population. The control group comprised more employees with a long job tenure (>20 years) than the intervention group. Means and standard deviations (SD) of the control group and intervention group at all measurements are presented in table 3. At baseline the intervention group scored higher on job crafting behavior, and lower on feelings of competence compared to the control group.

**Table 2:** Descriptive characteristics of control group and intervention group at T0. [SD=standard deviation.]

		Control group (26 schools; N=257)			Intervention group (4 schools; N=89)			Total (30 schools; N=346)	
		% ^a^	Mean ^b^ (SD)		% ^a^	Mean ^b^ (SD)		% ^a^	Mean ^b^ (SD)
Gender (female)		86.8			93.3			88.4	
Age (in years)			42.5 (11.80)			39.7 (12.06)			41.7 (11.91)
Function (teacher)		72.0			76.4			73.1	
Type of contract (permanent)		89.5			86.5			88.7	
Contract size (in hours per week)			26.5 (9.38)			27.0 (9.79)			26.6 (9.47)
Job tenure (years)									
	<1	8.6			13.5			9.8	
	1–5	25.3			28.1			26.0	
	5–10	12.5			19.1			14.2	
	10–20	32.3			30.3			31.8	
	>20	21.4 ^c^			9.0 ^c^			18.2	

^a^ Percentages are column percentages, and are tested with the Pearson χ^2^-test (horizontal comparisons). ^b^ Means are tested with the t-test. ^c^ P<0.05.

**Table 3:** Means and standard deviations (SD) of work stress, work stress determinants and level of implementation of the control and intervention group ^a^ at T0, T1 and T2.

		Control group			Intervention group	
		Mean (SD)	N		Mean (SD)	N
Emotional exhaustion (range: 1–7)						
	T0	2.58 (1.20)	257		2.61 (1.31)	89
	T1	2.43 (1.22)	265		2.57 (1.16)	85
	T2	2.55 (1.23)	265		2.49 (1.29)	54
Job crafting behavior (range: 1–5)						
	T0	3.83 (0.51) ^b^	257		3.97 (0.48) ^b^	89
	T1	3.77 (0.51) ^b^	265		3.93 (0.51) ^b^	85
	T2	3.77 (0.50)	265		3.87 (0.49)	54
Quantitative demands (range: 1–4)						
	T0	2.57 (0.61)	257		2.64 (0.54)	89
	T1	2.51 (0.58)	265		2.61 (0.56)	85
	T2	2.59 (0.59)	265		2.64 (0.59)	54
Emotional demands (range: 1–4)						
	T0	2.14 (0.54)	257		2.08 (0.48)	89
	T1	2.08 (0.53)	265		2.14 (0.54)	85
	T2	2.17 (0.54)	265		2.19 (0.57)	54
Unnecessary work tasks (range: 1–5)						
	T0	2.24 (0.75)	257		2.10 (0.77)	89
	T1	2.04 (0.82)	265		2.03 (0.72)	85
	T2	2.09 (0.79)	265		1.88 (0.85)	54
Autonomy (range: 1–3)						
	T0	2.56 (0.42)	257		2.49 (0.39)	89
	T1	2.60 (0.41) ^b^	265		2.47 (0.41) ^b^	85
	T2	2.52 (0.42)	265		2.54 (0.45)	54
Co–worker support (range: 1–5)						
	T0	4.31 (0.66)	257		4.27 (0.70)	89
	T1	4.30 (0.71)	265		4.31 (0.67)	85
	T2	4.23 (0.69)	265		4.40 (0.69)	54
Safe team culture (range: 1–5)						
	T0	4.05 (0.64)	257		4.05 (0.64)	89
	T1	4.07 (0.65)	265		4.04 (0.66)	85
	T2	3.97 (0.71)	265		4.15 (0.70)	54
Participatory leadership (range: 1–5)						
	T0	3.75 (0.78)	257		3.87 (0.64)	89
	T1	3.84 (0.74)	265		3.80 (0.71)	85
	T2	3.73 (0.80) ^b^	265		4.01 (0.73) ^b^	54
Quality of leadership (range: 1–5)						
	T0	3.69 (0.75)	257		3.72 (0.65)	89
	T1	3.78 (0.70)	265		3.88 (0.61)	85
	T2	3.60 (0.76) ^b^	265		4.02(0.56) ^b^	54
Feelings of competence (range: 1–7)						
	T0	4.08 (0.58) ^b^	257		3.90 (0.71) ^b^	89
	T1	4.05 (0.68)	265		3.96 (0.64)	85
	T2	4.09 (0.64)	265		4.06 (0.66)	54
Implementation process (range: 1–5)						
	T2	1.00 (0.00)	265		3.49 (0.72)	52
Implementation process items (range: 1–5):						
	Implemented actions (T2)	1.00 (0.00)	265		2.96(1.05)	52
	Information (T2)	1.00 (0.00)	265		3.83(0.79)	52
	Communication (T2)	1.00 (0.00)	265		3.58(0.10)	52
	Team commitment (T2)	1.00 (0.00)	265		3.27(0.82)	52
	Management commitment (T2)	1.00 (0.00)	265		3.96(0.84)	52
	Employee involvement (T2)	1.00 (0.00)	265		3.42(1.02)	52
	Participation in decision–making (T2)	1.00 (0.00)	265		3.12(1.02)	52

^a^ Differences between means of control group and intervention group are tested with the t-test. ^b^ P<0.05

### Effects related to condition

Results of the multivariate mixed model analyses are presented in table 4. No statistically significant intervention effect related to condition was found on emotional exhaustion (RQ1). This implies that there was no statistically significant difference between the intervention group and the control group on the level of emotional exhaustion at T2 as compared to T0. There was a statistically significant difference between the intervention group and the control group on leadership quality at T2 as compared to T0, in favor of the intervention group (β= -0.380) (RQ2). For the other work stress determinants, no intervention effects related to condition were found.

**Table 4:** Results of multivariate mixed model analyses, controlled for age, contract size and function. [CI=confidence interval; RQ=research question.]

	Time × Group ^a^				Time × Implementation ^b^		
	Regression coefficient (B)	95% CI	P-value		Regression coefficient (B)	95% CI	P-value
	**RQ1**				**RQ3**		
Emotional exhaustion	0.006	-0.345–0.357	0.974		-0.112	-0.266–0.043	0.155
	**RQ2**				**RQ4**		
Job crafting behavior	-0.091	-0.248–0.065	0.248		0.073 ^d^	0.007–0.139	0.032
Quantitative demands	-0.057	-0.250–0.135	0.553		-0.018	-0.095–0.060	0.652
Emotional demands	-0.013	-0.171–0.145	0.870		-0.001	-0.067–0.066	0.985
Unnecessary demands	0.238	-0.053–0.529	0.106		-0.125 ^d^	-0.243– -0.007	0.038
Leadership quality	-0.380 ^d^	-0.685 – -0.075	0.016		0.178 ^e^	0.053–0.302	0.006
Participatory leadership	-0.255 ^c^	-0.533–0.022	0.071		0.129 ^d^	0.014–0.244	0.028
Co-worker support	-0.079	-0.342–0.184	0.549		0.100 ^c^	-0.008–0.208	0.070
Autonomy	-0.006	-0.163–0.151	0.938		-0.038	-0.104–0.028	0.252
Safe team culture	-0.082	-0.334–0.170	0.518		0.113 ^d^	0.009–0.217	0.033
Feelings of competence	0.075	-0.124–0.274	0.454		0.048	-0.035–0.131	0.251

^a^ Time (T2 vs baseline) × group (control vs intervention) ^b^ Time (T2 vs baseline) × implementation ^c^ P<0.1. ^d^ P<0.05. ^e^ P<0.01.

### Effects related to implementation process

No statistically significant effects of the implementation process were found for emotional exhaustion (RQ3) and quantitative demands, emotional demands, autonomy and feelings of competence (RQ4). This implies that there was no statistically significant difference between employees in schools with high levels of implementation success compared to employees in schools with low levels of implementation success on these outcome measures at T2 as compared to T0.

However, statistically significant effects of the implementation process were found for unnecessary demands (β= -0.125), quality of leadership (β=0.178), participatory leadership (β=0.129), safe team culture (β=0.113) and for job crafting behavior (β=0.073) in the expected favorable direction. Employees in organizations with high levels of implementation process showed a more favorable change between T0 and T2 on these work stress determinants than employees in organizations with low levels of implementation.

## Discussion

The current study aimed to evaluate the effectiveness of a work stress prevention approach in primary education. When comparing intervention and control group, no effects of the approach on emotional exhaustion and most of the work stress determinants were found. However, results do show beneficial effects on quality of leadership. This is an important finding since it is known from literature that leadership is strongly related to work stress of subordinates (45). Furthermore, when taking into account the implementation process, results show that a high score on the implementation process (suggesting a more successful implementation process) was again associated with an increase in quality of leadership but also with a decrease in unnecessary demands and an increase in participatory leadership, safe team culture and job crafting behavior. These findings suggest that, when implemented successfully (that is, when employees are informed and involved, team and management is considered committed, and employees noticed actions or changes being implemented), the work stress prevention approach is potentially effective in decreasing work stress determinants.

There are several explanations for not finding statistically significant effects between the intervention and control group on emotional exhaustion and most of the work stress determinants. The COVID-19 pandemic (started after T1) affected the ability of schools to give priority to the action plans. Consequently, looking at the separate implementation process items, especially the score on the item regarding noticeable changes or actions being implemented as part of the approach was relatively low. The process evaluation demonstrated that the level of implementation of the approach varied greatly across the intervention schools and at some of the intervention schools, few actions were implemented (Bakhuys Roozeboom et al, 2023, submitted for publication). A low level of implementation of the approach obviously limited the effects the intervention was possible to bring about. Additionally, the response on the T2 questionnaire was relatively low affecting the statistical power to detect changes, which may also explain why overall effects of the approach on emotional exhaustion and most of the work stress determinants between the intervention group and control group could not be found.

Considering these circumstances, it is particularly interesting that effects on quality of leadership were found. From the results it is not clear what impacted the increase in (perceived) quality of leadership. This could be caused by the implemented actions, but it is also possible that employees have appreciated their leader taking part in the approach, and this positively impacted their perspective on quality of leadership. Either way this is an interesting finding, because besides their potential direct impact on employees’ wellbeing and stress, leaders also have an important role in organizational-level interventions (46). Since the work stress prevention approach is aimed to have a cyclical nature, the increase in quality of leadership may be a positive indicator of sustainable change.

Looking at the analyses that took into account the implementation process, as a proxy for the level of implementation (RQ3 and RQ4), results show that the level of implementation success does predict changes in the expected favorable direction on many of the work stress determinants. These findings suggest that, when implemented successfully (that is, when employees are informed and involved, team and management is considered committed, and employees noticed actions or changes being implemented), the work stress prevention approach is potentially effective in decreasing work stress determinants as identified in the logic model of change that may reduce emotional exhaustion in a longer term. Finding effects on secondary outcomes (work stress determinants), but not on primary outcomes (emotional exhaustion), appears to be a common phenomenon according to a recent review of reviews on organizational-interventions to improve the psychosocial work environment (14). A possible explanation for not finding a direct effect of the approach on emotional exhaustion could be related to the timing of the measurements. That is, to be able to detect effects on secondary as well as primary outcomes requires adequate timing of the measurements (47). However, optimal timing is often difficult to determine with these type of interventions, due to the fact that some effects of measures manifest themselves earlier than others. An additional follow-up measurement could be recommended to investigate longer-term effects of the approach, also on primary outcomes.

An important strength of the study is that in addition to per protocol analyses this study also researched the impact of implementation success on the effects of the approach. Although several researchers recommend these type of analyses, they are often lacking in effect evaluations (26). This study illustrates the importance of these type of analyses because they provide valuable additional information to draw conclusions on the effectiveness of interventions in relation to their implementation. Without these analyses, there is a risk of wrongly labeling interventions as not effective, while in practice they potentially are effective when implemented successfully.

Another strength of the study is that a logic model of change was built during the approach to select appropriate actions that targeted work stress as well as work stress risks. Consequently, the effect evaluation not only focused on effects of the approach on emotional exhaustion but also on specific work stress risks as determined in the logic model of change. This provided more insights into the mechanism of how the intervention works.

There are also some limitations that need to be considered. Since effects were hypothesized to occur at school level, data from new respondents were included in the analyses. This limited negative effects of drop-out (due to the long follow-up period between baseline and T1 and T2) on the statistical power to detect changes. However, the low response on the T2 questionnaire, did negatively affect the statistical power, and may also have resulted in a selection bias. Furthermore, the lack of randomization may have caused unknown confounders to be unevenly distributed across groups. The fact that intervention schools were the first to voluntarily apply for participation and that they scored higher on job crafting behavior at baseline, may indicate that these schools were more willing to address work stress and more open for change, which may have contributed to the study results. This is in line with what is already known from literature, namely that willingness to participate is an important prerequisite for organizational intervention to be successful.

Another limitation is the unevenly distributed number of schools in the intervention and control group. In the analyses to investigate the association between the level of implementation and progression of emotional exhaustion and work stress determinants between baseline and T2 (RQ3 and RQ4), the control group received the minimum score on implementation process scale (score=1). A disadvantage of this procedure is that the analyses are dominated by a large control group with a score of 1 (low variance). However, this procedure was chosen to maintain the same study population used to investigate RQ1 and RQ2. Moreover, this procedure makes optimal use of the power to detect changes.

### Concluding remarks

Despite the limitations the study has provided interesting insights. Although the study shows no direct effect of the approach on emotional exhaustion, results indicate that the approach has beneficial effects on (perceived) quality of leadership. In addition, results suggest that, when successfully implemented, the approach also has beneficial effects on several of the other work stress determinants. These results not only underline once more the importance of successful implementation of these kind of approaches, but also illustrate the need of including the level of implementation when studying the (potential) effectiveness of these type of approaches.
